# Data-driven risk assessment of the incursion of African swine fever virus *via* pig products brought illegally into South Korea by travelers based on the temporal relationship between outbreaks in China

**DOI:** 10.3389/fvets.2023.994749

**Published:** 2023-04-03

**Authors:** Dae-Sung Yoo, Ki-Hyun Cho, Seong-Keun Hong, Hae-Eun Kang, Jee-Yong Park

**Affiliations:** ^1^Veterinary Epidemiology Division, Animal and Plant Quarantine Agency, Gimcheon, Republic of Korea; ^2^College of Veterinary Medicine, Chonnam National University, Gwangju, Republic of Korea; ^3^Foreign Animal Disease Division, Animal and Plant Quarantine Agency, Gimcheon, Republic of Korea

**Keywords:** African swine fever, ASF, Bayesian, illegal importation, spatio-temporal, risk assessment

## Abstract

Since 2018, Asian countries have been affected by the African swine fever (ASF) virus, with major socioeconomic consequences. Moreover, the number of people traveling in Asian countries has been increasing, leading to an inevitable increase in the risk of ASF spread through livestock products carried by travelers. China and South Korea have close geo-economic ties and numerous international travelers. After the ASF outbreak in China in 2018, many illegally imported pig products (IIPPs) that were confiscated from travelers from China at the port of entry in South Korea tested positive for ASF. The detection of ASF virus (ASFV)-positive IIPPs highlights the need to further assess the risk of incursion by travelers and review the existing prevention strategies. Here, we investigated the temporal relationship between ASF outbreaks in China and the detection of ASFV-positive IIPPs in randomly confiscated samples from all ports of entry, such as flights and ships to South Korea, from 2018 to 2019 using a cross-correlation analysis. Based on the significantly correlated temporal lags between the bivariate time-series data, a risk assessment model, using the Bayesian framework, was built to estimate the distribution of the parameters for the risk assessment model and the monthly probability of ASF being introduced *via* IIPPs from China to South Korea. ASF outbreaks in China were significantly associated with the detection of ASFV-positive IIPPs in South Korea 5 months later. Hence, the monthly probability of ASFV-infected pig products imported from China *via* a traveler to South Korea was estimated to be 2.00 × 10^−5^, corresponding to a 0.98 mean monthly probability of at least one ASF-infected pig product arriving at ports of entry *via* travelers, from 2018 to 2019. To our knowledge, this study is the first attempt to estimate the risk of ASF introduction *via* pig products carried by international travelers to all ports from neighboring countries in the Asian region using commonly exchanged observed data. The data presented in this study can be used to refine the intervention strategies to combat the spread of transboundary animal diseases.

## Introduction

African swine fever (ASF) is a devastating porcine viral disease characterized by hemorrhagic fever, with virulent ASF virus (ASFV) strains causing up to 100% mortality. ASF was first reported in Kenya in 1921 (1) and has remained endemic in sub-Saharan African countries (2). The first transcontinental transmission of ASF from Western Africa to the Iberian Peninsula occurred in 1957 (2). It then spread to Europe and the Americas in 1999, apart from the island of Sardinia, Italy, where it has been in circulation since 1978 (2). The second transcontinental transmission began in Georgia in 2007 (3) and soon spread to Armenia, the Russian Federation, and Azerbaijan within a year (4). It has since continued to expand into many countries in Eastern Europe and entered several Western European countries. In August 2018, China was the first Asian country to report an outbreak of ASF. Neighboring Asian countries, including Mongolia, Vietnam, Cambodia, North Korea, Laos, Myanmar, Philippines, South Korea, Timor-Leste, and Indonesia soon reported outbreaks in 2019 (5). It has further propagated to other nations in Asia, including Papua New Guinea, India, Malaysia, Bhutan, Thailand, and Nepal (as of April 2022).

The first ASF outbreak in South Korean domestic pigs was reported on 16 September 2019, at a pig farm located in the northwestern border region, followed by 13 additional cases confirmed on 9 October 2019 (6). From 2020 to 2021, seven additional cases have been reported in the north-central and northeastern regions. ASFV infection in wild boars was first detected in the northwestern border region on 2 October 2019, and as of March 2022, more than 2,000 infected wild boars have been confirmed in four South Korean provinces in the northern and northeastern regions, while continuing to spread further south. Wild boars are considered a major risk factor and source of infection in pig farms in South Korea. In addition to wild boars, other potential sources of ASF include legally or illegally imported live pigs or pig products, food waste from international airplanes/ships, and fomites (7). Approximately 917.5 tons of illegally imported livestock products from countries with notifiable livestock infectious diseases were confiscated at all ports of entry to South Korea from more than 112,000 people in 2019 (8).

Specifically, pig products illegally brought into a country by international travelers are considered a major risk factor for transboundary transmission (9), particularly from neighboring countries that are likely to be socioeconomically interconnected. For example, more than 11.1 million travelers from China visited South Korea in 2019, according to the Immigration Statistics of Immigration Services in Korea (10). In addition, the commodities traded internationally between the two countries in 2019 yielded revenue of ~243 billion US dollars, making China the top-ranking country in terms of trade with South Korea (11).

African swine fever virus can survive for extended periods and is easily carried by travelers (12). ASFV is known to survive for at least 60 days in dry-cured products (13) as well as a type of blood sausage named sundae, in which the ASFV persistently survives for more than 1 year (9). In addition, pork sausage casings accounted for a high percentage of ASF-infected illegally imported pig products (IIPPs). In a previous experiment, pork sausage casings stored at room temperature were found to be positive for ASFV for 30 days (14). To mitigate this risk, various preventive measures were implemented in South Korea, including seizing pig products illegally brought in by travelers at international airports and ports. Moreover, some of these confiscated IIPPs were tested for ASF as part of a monitoring scheme implemented after the outbreak of ASF in China (15). Under this ASF monitoring scheme for IIPPs, multiple samples from confiscated pig products tested positive for ASF, underscoring the need for incursion risk assessment of pig products that are illegally brought by international travelers and the evaluation of the current prevention strategy based on these data. Moreover, because China reported the highest number of ASF outbreaks in the early stages of the ASF epidemic in Asia and produced ~50% of the total world pig production, it is very likely to affect neighboring countries such as Vietnam, Hong Kong, and Myanmar, which have experienced sequential ASF outbreaks (16).

This study aimed to explore the spatial and temporal relationship between the ASF situation in China and IIPPs brought in by passengers, using ASF notification data in China and data from a monitoring scheme for IIPPs in South Korea, followed by a risk assessment of ASF virus incursion using Bayesian inference from August 2018 to December 2020.

## Materials and methods

### ASF notification data

**Figure 1:**
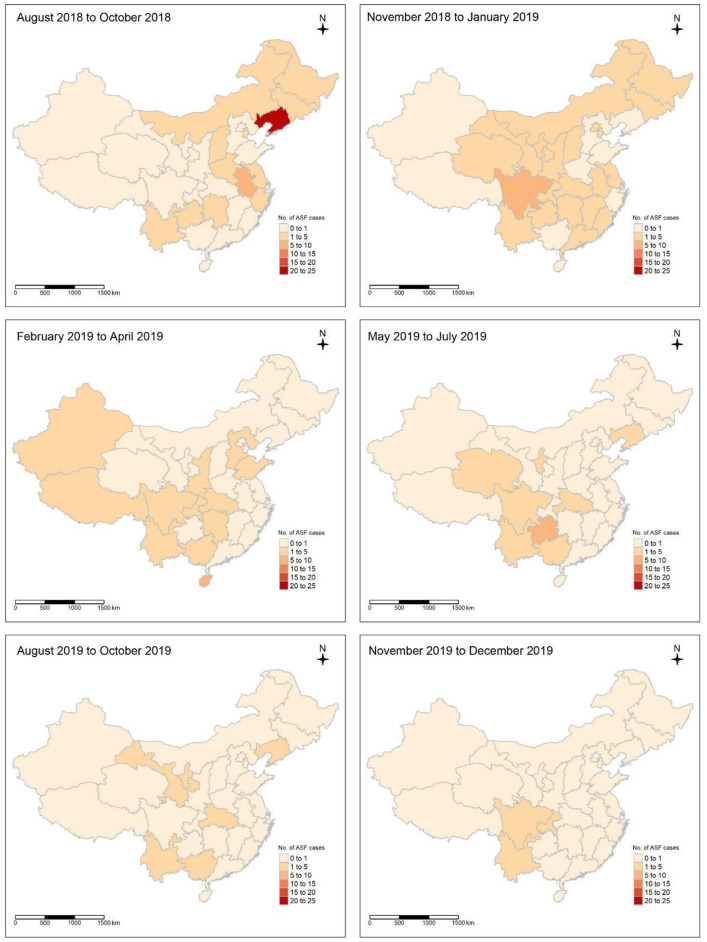
Spatial distribution of African swine fever (ASF) incidence in pig holdings from 2018 to 2019 in China. The cumulative number of ASF incidences at 3-month intervals is denoted by a color gradient with six equal intervals, where bright red represents the lowest-numbered class and dark red represents the highest-numbered class.

African swine fever notification data in China were collected from the World Animal Health Information System (WAHIS, https://wahis.woah.org/) of the World Organization for Animal Health [WOAH] (5). The data collection period ranged from 3 August 2018 to the date of the first ASF outbreak in Shenyang, China, to 31 December 2019 (17). This method helped minimize the interference from COVID-19, which heavily restricted international travel and impacted the incursion of the pathogen (18). During the study period, 178 cases were cumulatively reported in pig holdings in China (19). These data were aggregated into 34 province-level regions, including 23 provinces, five autonomous regions, four municipalities directly under the central government, and two special administrative regions, as shown in Figure 1.

### ASF monitoring data for illegally imported pig products

**Figure 2:**
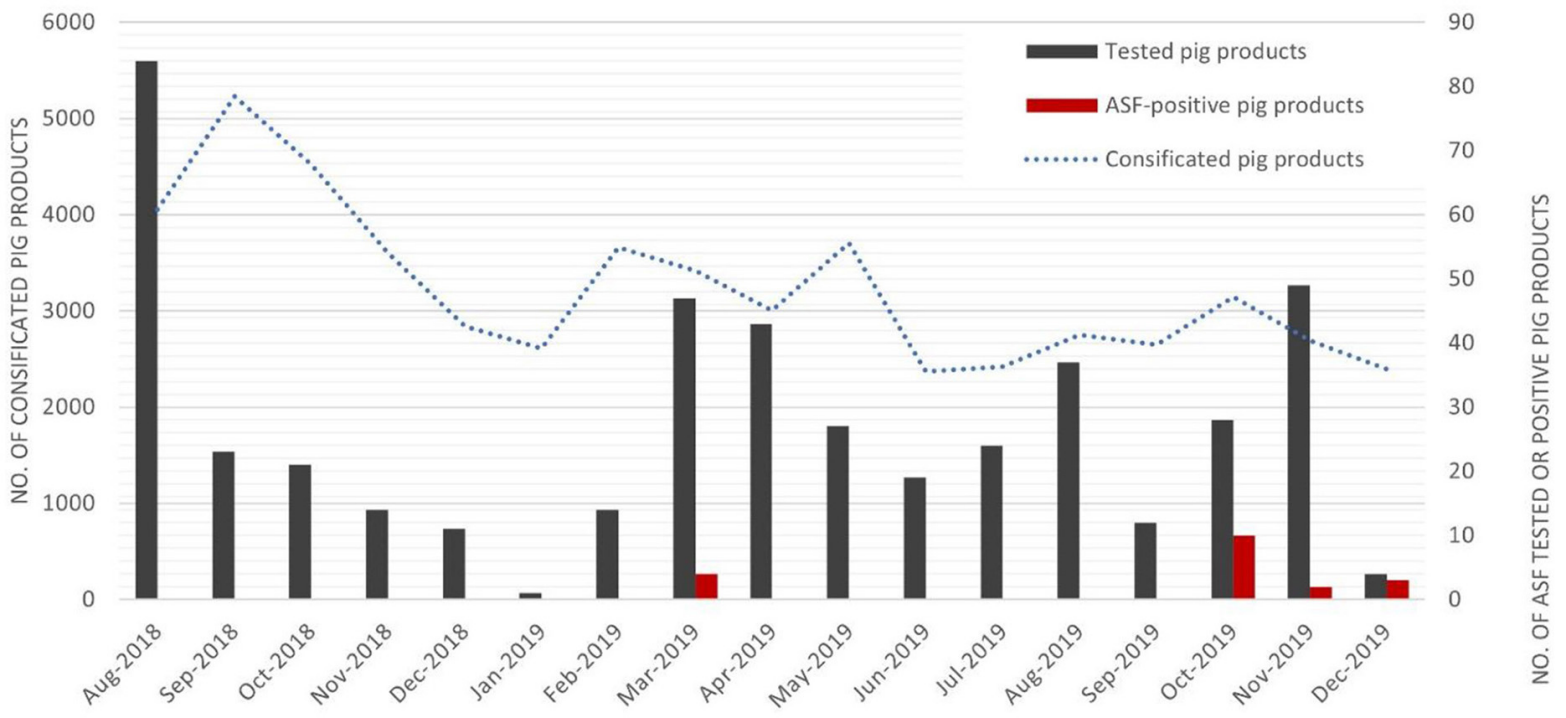
Monthly number of confiscated items (dotted blue line), samples tested for African swine fever (ASF) in confiscated pig products (black bar), and ASF virus-positive products (red bar) that were illegally imported from China, from August 2018 to December 2019 into South Korea.

As per the act on the prevention of contagious animal diseases, South Korea strictly prohibits the importation of all types of pig products containing bone, skin, internal organs, fat, and blood from ASF-infected countries. Active monitoring of ASF targeting IIPPs was implemented at all ports of entry to South Korea (*n* = 11) by the Animal and Plant Quarantine Agency (APQA) of South Korea. Under this scheme, IIPPs from travelers were confiscated and randomly sampled for ASF testing using a real-time polymerase chain reaction (PCR) test, as described in the terrestrial manual of the WOAH (20). This protocol was further reinforced after the first ASF outbreak was reported in August 2018 in China by increasing the frequency and sample size at each port of entry into South Korea. For example, 10 confiscated IIPPs from travelers originating from ASF-infected countries were randomly sampled weekly at Incheon International Airport (15), where a total of 173 flights, the largest number originating from China, were in operation every week in 2019 according to the 2019 air traffic statistics of Incheon International Airport (21). ASF monitoring data for IIPPs transported from China to South Korea from 3 August 2018 to 31 December 2019 were collected from the ASF monitoring scheme conducted by the APQA. The APQA data included the sampling date, type of product, weight, and country of origin. If the ASF virus was detected in the sampled IIPPs, the origin of flight or shipping of those products was further investigated. In total, 314 IIPPs were randomly sampled from confiscated items originating in China and tested using real-time PCR. A total of 34 IIPPs tested positive (Figure 2).

### Temporal correlation analysis between ASF outbreaks in China and ASFV-positive IIPPs in South Korea

Cross-correlation analysis was used to examine the temporal relationship between monthly ASFV-positive cases from IIPPs and monthly reported ASF cases in China. Cross-correlation represents the magnitude of the correlation between the bivariate time-series variables (i.e., the number of monthly ASF cases in pig holdings in China and ASFV-positive IIPPs in South Korea at different time intervals [lag or lead]). The cross-correlation of bivariate time-series variables at time lag k was tested under the null hypothesis of no cross-correlation between two variables using the Testcorr R package, version 0.2.0 in R software, version 4.1.1 (22). This package provided two testing procedures: one standard method and one robust method based on the types of test statistics. We employed the standard procedure with default settings to estimate the correlation coefficient and *p*-values (22). We used the time lag showing a significant correlation to adjust the time interval (i.e., months) between ASF cases in pig holdings in China and ASFV-positive IIPPs transported from China to South Korea for risk assessment.

### Risk assessment of the incursion of ASF virus through illegal importation by travelers from China

Given the time lag (*t*-lag) when a significant cross-correlation (*p*-value < 0.05) was observed between ASF cases in pig holdings in China and confiscation of ASFV-positive IIPPs in South Korea, we built the following mathematical formula to assess the incursion risk of ASF through pig products carried by travelers from China, *P*_*ASF*−*incursion, t*_, which denotes the probability that ASF-infected IIPPs were introduced into a port of entry in South Korea by one traveler in month *t*.


(1) $$\begin{array}{l} {\text{P}}_{\text{ASF}\;-\;\text{incursion},\;\text{t}}\;=\;{\text{P}}_{\text{slaughter},\;\text{t}\;-\;\text{lag}}\;\times \;{\text{P}}_{\text{illegal}\;\text{importation},\;\text{t}} \end{array}$$

where *P*_*slaughter, t*−*lag*_ represents the probability of one pig product containing the ASF virus and *P*_*illegal imporation, t*_ represents the probability of one pig product being illegally imported into South Korea. We assumed that the ASF virus was introduced in South Korea when the traveler from China illegally brought pig products at month *t*, specified by *P*_*illegal imporation, t*_, made of pigs originating from ASF-infected farms slaughtered at *t*-lag months before arrival month in South Korea, related to *P*_*slaughter, t*−*lag*_.

*P*_*slaughter, t*−*lag*_, was formulated by modifying the model proposed in previous studies (18, 23, 24), adding monthly ASF notification data on pig holdings in China and monitoring data of IIPPs in South Korea as follows:


(2) $$\begin{array}{l} {\text{P}}_{\text{slaughter}}\;=\;\frac{\text{Number}\;\text{of}\;\text{ASF}-\text{Infected}\;\text{pig}\;\text{farms}\times \text{H}\times \text{Infected}\;\text{period}\times \text{Prop}.\text{survival}}{\;\text{Number}\;\text{of}\;\text{Slaughtered}\;\text{pigs}\;\times {\text{P}}_{\text{notification}}} \end{array}$$

*The number of ASF-infected pig farms* is equal to the number of ASF-infected pig farms that reported their infection in China. *H* corresponds to the average herd size of the pig farms in China. In addition, *infected periods* refer to the duration during which ASF-infected farms slaughtered ASF-infected animals. *Prop.survival* is equal to the proportion of pigs that survived prior to the control measures. The *number of slaughtered pigs in China* is the number of pigs that are transported in abattoirs and slaughtered in China. *P*_*notification*_ denotes the probability of ASF notifications for pig farms in China. *P*_*notification*_ was parameterized by estimating the posterior distribution.

*P*_*slaughter*_ is related to the proportion of ASF-infected pigs slaughtered for consumption despite the use of control interventions. In other words, it refers to the probability of one pig product containing the ASF virus, as ASF-infected pig farms slaughtered their infected pigs without notification to a competent authority. This notion can be translated into the mathematical formulation as follows:


(3) $$\begin{array}{l} \begin{array}{l} \text{True}\;\text{number}\;\text{of}\;\text{ASF}\;\text{infected}\;\text{farms}\;\times \;\text{H}\\ \;\times \;\text{Infected}\;\text{periods}\;\times \;\text{Prop}.\text{survival} \end{array} \end{array}$$

where the *true number of ASF-infected farms* corresponds to the actual number of ASF-infected farms, both with and without notifications to a competent authority. The total number of ASF-infected pigs per ASF-infected farm that were transported to a slaughterhouse and processed into pig products was derived by multiplying infected periods with *H* and *Prop.survival*. Consequently, the total number of ASF-infected pigs processed into pig products in China was derived by multiplying them by the *true number of ASF-infected farms*. In such a context, the preceding equation yielded the total number of ASF-infected pigs that survived and were slaughtered in China. For this equation, the *true number of ASF-infected farms* was required to estimate the proportion of ASF-infected pigs slaughtered for consumption. The *true number of ASF-infected farms* was regarded as an unknown variable, but could be expressed using the number of newly reported ASF-infected farms, which was an observed variable (i.e., ASF notification data from WOAH), given by the following equation:


(4) $$\begin{array}{l} \text{True}\;\text{number}\;\text{of}\;\text{ASF}-\text{infected}\;\text{farms}\times {\text{P}}_{\text{notification}}\;\\ \;=\text{Number}\;\text{of}\;\text{ASF}\;\text{infected}\;\text{pig}\;\text{farms} \end{array}$$

Because the number of ASF-infected pig farms reporting their infection to a competent authority was a result of the reported proportion of the true number of ASF-infected farms, the true number of ASF-infected farms was replaced with the *number of ASF-infected pig farms/P*_*notification*_, just as in the first equation for *P*_*slaughter*_. Subsequently, for monthly risk assessment, the number of ASF-infected pig farms that reported their infection was converted into the monthly number of newly reported ASF-infected farms in China. Then, we divided it by the monthly number of slaughtered pigs in China to reformulate the probability of one pig product containing the ASF virus in each month as follows:


(5) $$\begin{array}{l} {\text{P}}_{\text{slaughter}}\;=\;\frac{\text{Monthly}\;\text{number}\;\text{of}\;\text{ASF}\;\text{infected}\;\text{pig}\;\text{farms}\;\times \text{H}\times \text{infected}\;\text{periods}\times \;\text{Prop}.\text{survival}\;}{\text{monthly}\;\text{number}\;\text{of}\;\text{slaughtered}\;\text{pigs}\;\times {\text{P}}_{\text{notification}}} \end{array}$$

For the data-driven risk assessment modeling approach, we used the ASF notification data in China from the WOAH (5, 24) as the input for the monthly number of newly reported ASF cases in pig farms and the *monthly number of ASF-infected pig farms*. We parameterized *P*_*notification*_ as an unknown variable for which the posterior distribution was estimated. The rest of the elements included in *P*_*slaughter*_ were assigned specific values and assumed to be constant and deterministic based on previous studies (Table 1). For example, *H*, the average number of herds in pig farms in China, was set to 1,644 based on the 2019 report of the WOAH (24, 25). The *infected period* on a monthly scale was set to 0.63 because the previous study established the *P*_*slaughter*_ equation (24), and the *infected period* per year was set to 0.63 (i.e., 230/365). We used the same value for infected periods on a monthly scale because the time of the year a farm slaughtered infected animals was consistent between monthly segments. *Prop.survival*, the proportion of ASF-infected pigs surviving the infection and being slaughtered, was set to 0.36, as specified in a previous study (24, 26). A total of 54,419,200 pigs/12 months, according to the China Animal Husbandry Yearbook, 2020, (24, 27) was used as the *monthly number of slaughtered pigs* in China.

**Table 1:** Input variables of the risk assessment model for the incursion of African swine fever through illegal importation of pig products from China.

**Variables**	**Description**	**Values (time scale)**	**Source**
No. of ASF-infected farms	Monthly number of ASF-infected pig farms	Variable (monthly basis)	(5)
*H*	Average number of herds in ASF infected-pig farms	1,644 (constant)	(25)
Infected periods	Duration of ASF infection in months	0.63 (constant)	(24)
Prop.survival	Proportion of ASF-infected pigs that survived the infection and were slaughtered	0.36 (constant)	(26)
Monthly number of pigs slaughtered	Monthly number of pigs slaughtered in China	54,419,200/12 (constant)	(27)
*P_notification*	Probability of notification of ASF infection	Parameterized	
*P_illegal importation*	Probability of pig products being illegally imported from China	Parameterized	
*P_confiscated*	Probability of illegally imported pig products being confiscated at the port of entry	Parameterized	
*N_*samples*_*	Monthly number of samples from confiscated pig products	Variable (monthly basis)	(8)
*N_*travelers*_*	Monthly number of incoming travelers to South Korea from China	Variable (monthly basis)	(10)

Next, to derive *P*_*illegal imporation, t*_, the probability of one pig product being illegally imported into South Korea at month *t*, we assumed that the probability of the APQA confiscating ASF-infected pig products following illegal importation by a traveler, *P*_*ASF*−*positive IIPPs*_, was the mathematical product of three independent probabilities: (i) the probability of one pig product containing the ASF virus (*P*_*slaughter*_), which was pre-defined, (ii) the probability of one pig product being illegally imported into South Korea (*P*_*illegal importation*_), and (iii) the probability of one IIPP being confiscated at the port of entry (*P*_*confiscated*_). By multiplying these three probabilities, the probability of one confiscated IIPP testing positive for ASF (*P*_*ASF*−*positive IIPPs*_) was yielded as follows:


(6) $$\begin{array}{l} {\text{P}}_{\text{ASF}-\text{positive}\;\text{IIPPs}}={\text{P}}_{\text{slaughter}}\times {\text{P}}_{\text{illegal}\;\text{importation}}\times {\text{P}}_{\text{confiscated}} \end{array}$$

To incorporate the significantly correlated time lag between ASF cases in pig holdings in China and ASFV-positive IIPPs in South Korea into the risk assessment model, which was identified in the temporal correlation analysis, we presumed that the proportion of ASF-infected pigs slaughtered for consumption in China at month *lag* before month *t*, *P*_*slaughter, t*−*lag*_, determined by *P*_*notification, t*−*lag*_, influenced the ASF incursion risk *via* the incoming travelers from China to South Korea at month *t*, *P*_*ASF*−*positive IIPPs, t*_, with illegal importation, *P*_*illegal importation, t*_, and confiscation, *P*_*confiscated, t*_ taking place in South Korea at month *t*. In this context, the equation can be rewritten with different notations, as follows:


(7) $$\begin{array}{l} P_{ASF-positive\;IIPPs,t}=P_{slaughter,\;t-lag}\times P_{illegal\;importation,t}\times \;\\ P_{confiscated,t} \end{array}$$

Since *P*_*illegal importation, t*_ and *P*_*confiscated, t*_ are unknown variables, such as *P*_*notification, t*−*lag*_, we parameterized them and estimated their posterior marginal distribution using Bayesian inference based on previous studies, as shown in Table 1. For the parameter estimation of those three variables with the Bayesian framework, we used binomial likelihood to model the monthly number of ASFV-positive IIPPs as a function of *P*_*ASF*−*positive IIPPs, t*_, the product of three components and the number of IIPPs randomly sampled for the test at month *t*, *N*_*samples, t*_.


(8) $$\begin{array}{l} \text{Monthly}\;\text{number}\;\text{of}\;\text{ASF}-\text{positive }results\text{ in}\;\text{IIPPs},\text{t}\;\sim \;\\ \;\text{Binomial}\;({\text{N}}_{\text{samples},\text{t}},{\text{P}}_{\text{ASF}-\text{positive}\;\text{IIPPs},\text{t}}) \end{array}$$

Because the binomial likelihood function is composed of the number of independent trials and discrete probability distribution of the number of successes, the actual number of samples at month *t*, *N*_*samples, t*_, compiled from the monitoring data of APQA, South Korea (8), was assigned to the trials and *P*_*ASF*−*positive IIPPs, t*_was assigned to the probability of success. Then, the posterior distribution for the three parameters, *P*_*notification, t*−*lag*_, *P*_*illegal importation, t*_, and *P*_*confiscated, t*_ was estimated using the Bayesian framework with the No-U-Turn sampling method through 32,000 iterations (four chains with 10,000 draws, burn-in of 2,000 per chain) (28). The prior distributions of two parameters (i.e., *P*_*illegal importation*_ and *P*_*confiscated*_) were specified by a non-informative prior, whereas, for *P*_*notification*_, a beta distribution was assigned, based on previous studies about ASF import risk assessment (24, 29). Convergence of sampling for posterior distribution was diagnosed by the Gelman–Rubin index (<1.05). With the posterior distribution estimates of three parameters, the probability that ASF-infected IIPPs were introduced into a port of entry in South Korea by one traveler before confiscation by the APQA (*P*_*ASF*−*incursion, t*_) at month *t* was computed as the proxy of ASF incursion risk. As a result, we calculated the monthly probability of at least one ASF-infected pig product being introduced into South Korea by all travelers from China at month *t* using the monthly number of incoming travelers to South Korea (*Ntravelers*), extracted from the Immigration statistics of the Korean Immigration Service (10), based on the previous study (29), as follows:


(9) $$\begin{array}{l} \text{P}(\text{monthly}\;\text{number}\;\text{of}\;\text{ASF}-\text{infected}\;\text{IIPPS}\geq 1)=\;\\ \;1-{\left(1-{\text{P}}_{\text{ASF}-\text{incursion},\text{t}}\right)}^{\text{Ntravelers},\text{t}} \end{array}$$

In this model framework, we reported the posterior distribution of three parameters of the risk assessment model, *P*_*notification*_, *P*_*illegal importation, t*−*lag*_, and *P*_*consificated, t*_, together with the monthly probability of at least one ASF-infected pig product introduced into South Korea through travelers from China. The Pymc3 package in Python software version 3.6.1 software was used to conduct Bayesian inference on the parameters of the risk assessment model (30).

## Results

### Temporal correlation between ASF outbreaks in China and IIPPs in South Korea

**Figure 3:**
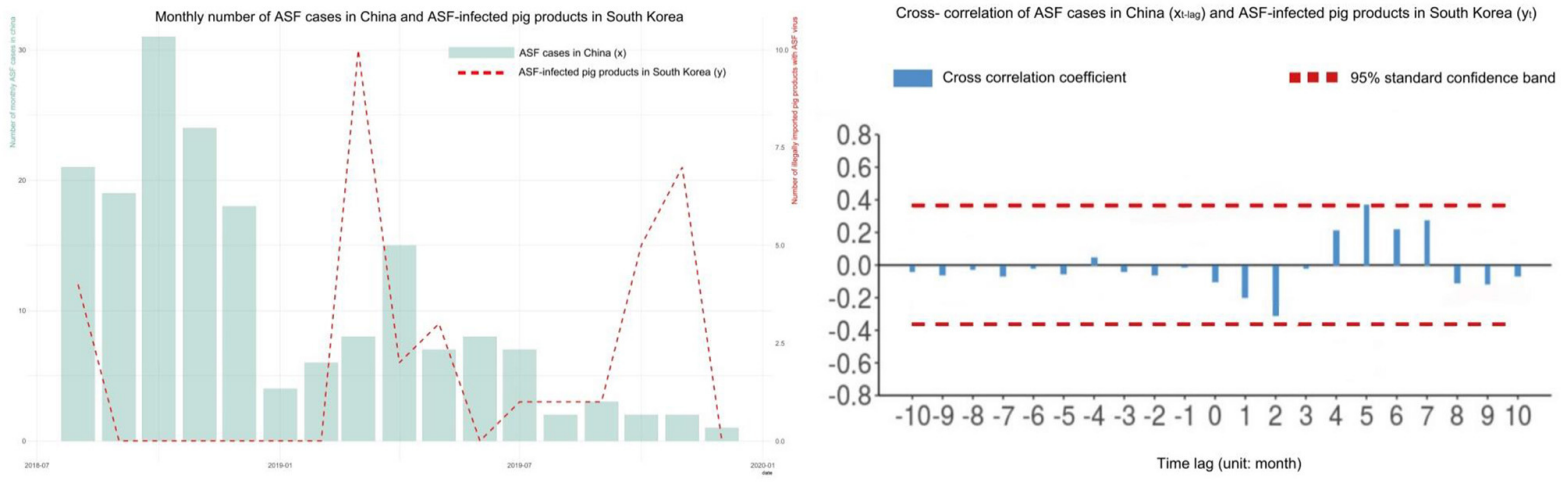
Monthly distribution of African swine fever (ASF) cases reported in pig farms, China, ASF virus-infected pig products illegally imported into South Korea **(left)**, and cross-correlation plot between the bivariate variables **(right)**.

Figure 3 illustrates the monthly distribution of ASF cases on pig farms in China and ASFV-positive IIPPs in South Korea, together with a cross-correlation plot between the two variables. ASF outbreaks in China have reported a higher number of monthly cases in the initial phase of the epidemic. The epidemic curve exhibited two peaks, with the highest number of cases in October 2018 and a relatively lower number of cases in April 2019. Similarly, there were two elevations in ASFV-positive IIPPs in South Korea, in March and November 2019. In addition, ASFV-positivity (the number of ASFV-positive IIPPs divided by the number of samples of IIPPs) of the monitoring data of IIPPs from China showed a comparable temporal pattern (data not shown). ASF outbreaks in China were significantly correlated with ASFV-positive IIPPs in South Korea 5 months later (correlation coefficient = 0.37, *p* = 0.045).

### Inference of the probability of ASF-infected pig products being illegally imported to South Korea

Based on Bayesian inference for the posterior distribution of the three parameters of the risk assessment model, the mean of the probability of notification of ASF, *P*_*notification*_, was estimated to be 0.401 (95% highest density interval [HDI] = 0.246–0.556), the mean of the probability of pig products illegally imported from China (*P*_*illegal importation, t*−*lag*_) was 0.032 (95% HDI = 0.012–0.054), and the mean of the probability of IIPPs being confiscated at the port of entry (*P*_*consificated, t*_) was 0.499 (95% HDI = 0.189–0.817).

**Figure 4:**
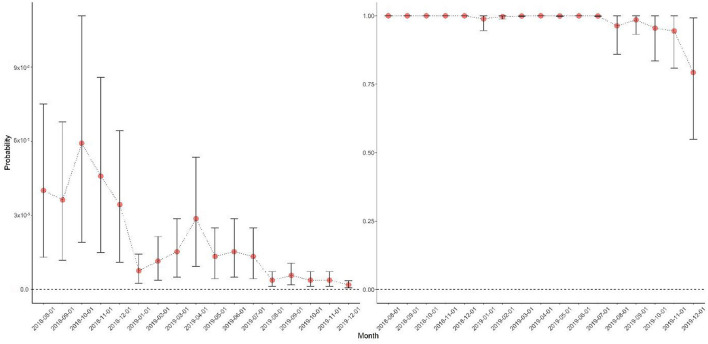
Monthly risk estimates of the incursion of African swine fever (ASF) through illegal importation of pig products from China. The **(left)** panel illustrates the probability that ASF-infected illegally imported pig products (IIPPs) by one traveler were introduced into a port of entry in South Korea before confiscation, and the **(right)** panel represents the probability of at least one ASF virus-infected pig product being introduced into South Korea. The red circle denotes the mean of probability, with the black error bar representing 95% of the highest density interval.

Given the estimates and the monthly volume of incoming travelers from China to South Korea, the incursion risk of the ASF virus into South Korea *via* IIPPs was quantified, as shown in Figure 4. The monthly probability of at least one ASF-infected pig product being introduced into South Korea through travelers from China was >0.79 from August 2018 to December 2019. Despite a significant decrease in the *P*_*ASF*−*incursion, t*_ after 2019, the probability of at least one ASF-infected pig product being introduced into South Korea remained high until August 2019. However, this probability decreased considerably in the fourth quarter of 2019, as the number of travelers decreased.

## Discussion

Many Asian countries are confronted with the growing risk of ASF incursions as outbreaks become widespread in geographically and socioeconomically interconnected countries (31). The sharp decline in pig supply due to the massive depopulation of pigs in infected premises could facilitate not only international meat trade but also illegal importation across the border, contributing to ASF transmission internationally (12). In this context, as China and South Korea exhibit spatiotemporally close relationships in terms of ASF in pigs and pig products, we evaluated the risk of ASF incursion *via* illegal importation of pig products from China to South Korea based on relevant data rather than empirical assumptions to validate our estimates.

In this study, the cases of ASFV-positive IIPPs exhibited a similar temporal pattern, with a lag behind, as the ASF notifications in pig farms in China. This can be partly explained by the changes in the epidemic severity in China and the temporal variation in the volume of travelers to South Korea. As previously mentioned, ASF in pigs in China rapidly increased in August 2018, and the peak of the outbreak was observed 2 months later. Thus, the risk of ASF-infected pigs being slaughtered without timely intervention was likely to be very high during this period. However, there are also time requirements for pork and pig products to reach consumers. For example, after slaughter, ASF-infected pigs are routinely processed into different types of products, such as sausages and pig jerky, requiring various lengths of time to finish processing. It can take 3–15 days to produce sausages (32). The shelf life of products in the market and the storage time can also contribute to the time lag. Indeed, ~52% of the ASFV-positive IIPPs examined in this study were sausages. This could explain why the first ASF cases in IIPPs were detected in countries neighboring China during similar months. For instance, Japan and Taiwan reported ASFV-positive IIPPs from China on 23 October 2018 and 20 November 2018, respectively (33, 34).

The IIPPs confiscated by the monitoring scheme in South Korea were positive for ASF by real-time PCR, but no viable ASFVs were detected. However, the ASFV-positive IIPPs from China included dry-cured products, and the ASFV has been reported to survive for at least 60 days (13), and in a variety of blood sausages, the ASFV can survive for more than 1 year (9). Moreover, pig sausage casings, which account for a high proportion of ASFV-positive IIPPs, have been reported to retain ASFV for 30 days when stored at room temperature (14).

The number of travelers from China to South Korea also increased in February and March, which is consistent with the peaks in the number of ASFV-positive IIPPs. During this period, most workers and students came to South Korea before the spring semester started, and the job market was wide open (10). As a result, the 5-month time gap between the ASF outbreaks in China and the number of ASFV-positive IIPPs in South Korea could be attributed to the increased influx of travelers from China and the duration associated with the supply chain of pig products.

According to the Korean Immigrant Office (10), on average, 0.96 million travelers (sum = 16.31 million) visited South Korea from China monthly from August 2018 to December 2019. During the same period, APQA confiscated 54.89 tons of IIPPs from travelers from China. Based on these data and the inference on the proportion of ASF notifications, the probability of pig products being illegally imported and IIPPs being confiscated at a port of entry—the monthly probability that ASF-infected pig products originating from China were illegally imported into South Korea—was estimated to be more than 0.79 from 2018 to 2019. This result was relatively higher than the result from a risk assessment conducted in Japan, where the mean probability of ASF virus incursion *via* pig products from China was estimated to be 0.697 (29). Another recent risk assessment reported that the mean annual probability of ASF virus introduction through pig products brought by air passenger luggage from China to the United States was 0.428 (35). This was almost two times as high as previous estimates, owing to the growing number of cases in Asia (36). Moreover, the three estimates of the risk assessment model (*P*_*notification, t*−*lag*_, *P*_*illegal importation, t*_, and *P*_*confiscated, t*_) were comparable to the distributions of the corresponding input variables used in previous studies (24, 29). However, it is difficult to compare the results directly with those of other studies because of different evaluation methods. In addition, the annual number of incoming travelers to South Korea from China (*n* = 12.28 million) was twofold higher than that in a previous study (*n* = 5.95 million). This resulted in a high-risk estimate for at least one ASF-infected pig product being introduced into South Korea.

A limitation of this study is that we formalized the risk assessment model in a simple manner while employing relatively few parameters because the posterior distribution of parameters by Bayesian inference can only be estimated using the available dataset. We also employed a Bayesian framework, which allowed the estimation of parameter distribution with uncertainty, given the small sample size. Indeed, 314 samples of IIPPs from China were subjected to ASF testing from 3 August 2018 to 31 December 2019, equivalent to 18.47 per month. We utilized only the results of the temporal analysis (i.e., cross-correlation analysis) to develop the risk assessment model. Because geographical information about the origin of all IIPPs sampled for the test was not available, we could not undertake province-specific risk assessment or consider the variation of input variables across the provinces, such as the probability of ASF notifications in pig holdings and the number of travelers at the province level. Thus, a model that accommodates regional disparities should be designed in the future.

Nonetheless, to our knowledge, this study represents the first attempt to estimate the risk of ASF-infected IIPPs carried by travelers arriving at all ports of entry from neighboring countries using monthly monitoring and outbreak data. Our data can provide practical insights into the monthly risk variation of the spread of ASF, which can assist countries in establishing preparedness and prevention strategies.

## Data availability statement

The original contributions presented in the study are included in the article/supplementary material, further inquiries can be directed to the corresponding author.

## Author contributions

D-SY and K-HC conceived and designed the study and drafted the original manuscript. D-SY, K-HC, S-KH, H-EK, and J-YP acquired the data. J-YP and S-KH supported data collection. D-SY performed data analysis. J-YP and H-EK edited the manuscript drafts. All authors read and approved the final manuscript.
